# Burden of congenital and hereditary anomalies and their epidemiological attributes in the pediatric and adult population of Peshawar valley, Pakistan

**DOI:** 10.12669/pjms.40.10.9234

**Published:** 2024-11

**Authors:** Syeda Farwa Naqvi, Umi Ameena, Waheed Uddin Qazi, Salman Ahmad, Anjum Iqbal, Sajid Malik

**Affiliations:** 1 Syeda Farwa Naqvi, Human Genetics Program, Department of Zoology, Quaid-i-Azam University, Islamabad, Pakistan; 2 Umi Ameena, Human Genetics Program, Department of Zoology, Quaid-i-Azam University, Islamabad, Pakistan; 3 Waheed Uddin Qazi, Human Genetics Program, Department of Zoology, Quaid-i-Azam University, Islamabad, Pakistan; 4 Salman Ahmad Department of Zoology, Kohat University of Science and Technology, Kohat, Pakistan; 5 Anjum Iqbal, Human Genetics Program, Department of Zoology, Quaid-i-Azam University, Islamabad, Pakistan; 6 Sajid Malik, Human Genetics Program, Department of Zoology, Quaid-i-Azam University, Islamabad, Pakistan

**Keywords:** Birth defects, Neurological disorders, Limb defects, Genetic load, Consanguinity, Khyber Pakhtunkhwa

## Abstract

**Objectives:**

To elucidate the burden and clinico-epidemiological attributes of congenital and hereditary anomalies (CA) in the extended Peshawar Valley of Pakistan.

**Method:**

This is a multicenter cross sectional study carried out in Buner, Charsadda, Mardan, Nowshera, Peshawar and Swabi districts, during 2017-2021. The pediatric and adult patients with CA were recruited from hospitals, public places and through door-to-door surveys. The anomalies were classified with the help of specialized clinicians. Descriptive statistics was employed.

**Results:**

In this study, 1065 independent subjects with certain types of CA from independent households were included. The index males were 71%; the majority of subjects originated from rural areas (72%), and spoke *Pashto* (96%). The CA were categorized into 10 major and at least 104 minor categories. There was highest representation of neurological disorders, n=375 (proportion.: 0.352; 95% CI: 0.323-0.381), followed by limb defects (n=281; prop.: 0.264), sensorineural defects (n=128; prop.: 0.120), musculoskeletal defects (n=84; prop.: 0.079), visual impairments (n=67; prop.: 0.063), hemoglobinopathies (n=40; prop.: 0.038), ectodermal disorders (n=34; prop.: 0.032), cardiovascular anomalies (n=19; prop.: 0.018), and orofacial anomalies (n=19; prop). Among the neurological disorder, intellectual disabilities and cerebral palsy were highly prevalent. The majority of the cases had a sporadic presentation (68%), and isolated occurrence (72%), whereas parental consanguinity was witnessed in 58% of cases.

**Conclusion:**

A wide range of CA were witnessed in this cohort with a preponderance of neurological disorders. The majority of the anomalies are of severe nature rendering a high morbidity burden in the population and requiring early detection, intervention and management.

## INTRODUCTION

Hereditary and congenital anomalies (CA) are one of the major infant and childhood problems.^1^ Approximately eight million births with a certain type of serious birth defects occur worldwide annually, the majority of which are documented in middle-income and underdeveloped countries.^2^ One of the leading causes of perinatal and infant morbidity and mortality are CA.

Considerable geographic and ethnic variations can be observed in the incidence, frequency and distribution of CA. It is reported to be very low as 1.1% in Japan and 4.3% in Taiwan.^3^ In India, birth defect prevalence varies from 61-70/1000 births.^4^ CA of severe nature cause mortalities, life-long disabilities which significantly compromise the quality of life of the subject and his/her family.^3^

Due to various reasons, families with low socio-economic conditions have a more frequent occurrence of CA. The incidence of CA is influenced by maternal exposure to pesticides, chemicals, drugs, tobacco, alcohol and radiation, and nutritional status during pregnancy.^3^ One of the most important factors known to be associated with the increased incidence of CA is consanguineous marriages which are deeply rooted in Muslim countries.^5^,^6^ The parental consanguinity almost doubles the risk of CA including metabolic disorders, blindness, deaf-muteness, and intellectual disability.^7^,^8^

Owing to its unique biodemographic background Pakistan faces a high burden of CA.^9^-^12^ Studies in rural Pakistan have shown that affected individuals with CA are misdiagnosed and mishandled, leading to high levels of parental anxiety as well as a trench in family resources.^13^ While several studies have reported CA in neonates, the nature and pattern of CA in young and adult populations remain unelucidated. To this end, to elucidate the burden and prevalence-pattern of CA in the population of Peshawar Valley, Pakistan, the present clinico-epidemiological study was carried out. This is a pilot study reporting large first-hand data and focusing the pediatric and adult population strata.

## METHODS

The Peshawar Valley is a broad terrain stretching in the central part of the Khyber Pakhtunkhwa (KP) province of Pakistan. It lies on the eastern side of the Khyber Pass that connects Pakistan to Afghanistan. The total population residing in Peshawar Valley is 11.4 million.^14^ Peshawar the capital city of KP, and is the 9^th^ largest metropolitan city of Pakistan. Commonly inhabiting ethnicities are Pashtun and Hindkowans.

### Sampling, classification and definitions of anomalies:

A cross-sectional study was carried out during 2017-2021 in the extended Peshawar Valley of Pakistan.

The subjects/families were recruited through visits to district headquarters hospitals (DHQ), community centers, and random door-to-door surveys through convenience sampling. This study is a part of a larger effort to elucidate the prevalence-pattern of CA in KP province.^11^,^15^,^16^ Formal consent was obtained from the family head/guardian and the consent approval was facilitated by the field resource person or lady-health-visitors.

### Ethical Approval:

The study protocol was approved by the Ethical Review Committee of Quaid-i-Azam University (DAS-15-, June 3, 2015). The study was conducted in compliance with international guidelines for human research protection, including the principles outlined in the contemporary revision of the Declaration of Helsinki.

The cross-sectional reporting guideline of the Strengthening the Reporting of Observational Studies in Epidemiology (STROBE) statement was used.^17^ The phenotypic detail and information on biodemographic variables were recorded on a structured proforma. The affected individuals were physically examined with the help of resident medical officers and specialized doctors at the respective DHQ. The anomalies were primarily diagnosed and categorized by the medical specialist and were further classified using ICD-10 (https://icd.who.int/browse10/2019/en) and OMIM (www.omim.org/) databases. Previous medical record when available was taken into account. For each subject, a detailed pedigree up to three generations was constructed and information on family history, sporadic/familial nature, disease segregating generations, isolated/syndromic condition and parity were obtained. Anomalies with traumatic, acquired or pathogenic origin were not included.

### Statistical analysis:

The distribution of CA was assessed across the biodemographic variables of the index cases. Descriptive summaries were generated. The significance of random distribution was assessed through Chi-test, Fisher exact test and T-test implemented through GraphPad Prism Software.

## RESULTS

### Sample characteristics:

**Fig.1:**
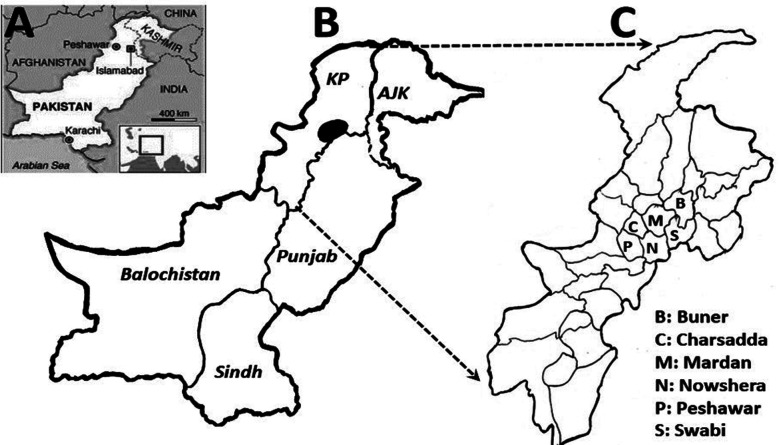
Map of Pakistan (A-B) with superimposed map of KP (C) depicting sampling districts of extended Peshawar valley.

A total of 1065 index subjects (males 752; females 313) from independent families were recruited. The mean age of subjects was 16.9±12.9 years and the pediatric population (up to 18 years) comprised 66% (n=703) of the sample. There were participants from six districts (Table-I; Fig.1). The majority of subjects originated from rural areas (72%), spoke *Pashto* (96%), and belonged to Pathan and Khattak caste-systems (19% and 17%, respectively). The subject’s literacy was 43%, and mostly belonged to low or low-middle socio-economic class (38% and 22%, respectively).

**Table-I:** Demographic attributes of index males and females.

Variable	Index male		Index female		Total	

	No.	%	No.	%	No.	%
District*						
Nowshera	225	71	91	29	316	30
Peshawar	195	73	72	27	267	25
Swabi	161	69	74	31	235	22
Mardan	84	76	27	24	111	10
Buner	59	58	43	42	102	10
Charsadda	28	82	6	18	34	3
Sum	752	71	313	29	1,065	100
***Rural/urban origin***						
Rural	534	69	237	31	771	72
Urban	218	74	76	26	294	28
***Language***						
Pashto	725	71	301	29	1,026	96
Hindko	17	61	11	39	28	3
Urdu	10	91	1	9	11	1
Age ranges (years)*						
Up to 9	239	65	126	35	365	34
>9-19	262	73	96	27	358	34
>19-29	118	70	50	30	168	16
>29	133	76	41	24	174	16
Caste-system*						
Pathan	125	61	79	39	204	19
Khattak	118	66	61	34	179	17
Yousafzai	118	83	25	17	143	13
Afghan	32	84	6	16	38	4
Awan	22	65	12	35	34	3
Momand	21	66	11	34	32	3
Khalil	24	80	6	20	30	3
Others	276	72	105	28	381	36
Literacy (age >5 years)*						
Illiterate	319	69	142	31	461	57
Literate	267	76	85	24	352	43
Primary	135	77	41	23	176	50
Middle	52	76	16	24	68	19
Secondary	60	80	15	20	75	21
Graduate and higher	20	61	13	39	33	9
***Economic quintile (PKR)***						
Poor (up to 50K)	139	71	57	29	196	18
Low (50-70K)	290	71	118	29	408	38
Low-middle (70-100K)	177	74	61	26	238	22
Middle (100-150K)	67	60	45	40	112	11
High-middle (>150K)	79	71	32	29	111	10

* Differences in the distribution were statistically significant.

### Diversity of CA:

The CA observed in the included individuals were classified into ten major and at least 104 minor categories (Table-II). There was highest representation of neurological disorders, n=375 (proportion.: 0.352; 95% CI: 0.323-0.381), followed by limb defects, n=281 (prop.: 0.264), sensorineural defects, n=128 (prop.: 0.120), musculoskeletal defects, n=84 (prop.: 0.079), visual impairments, n=67 (prop.: 0.063), hemoglobinopathies, n=40 (prop.: 0.038), ectodermal disorders, n=34 (prop.: 0.032), cardiovascular anomalies, n=19 (prop.: 0.018), and orofacial anomalies, n=19 (prop.: 0.018).

**Table-II:** Major and minor categories of CA.

Major/minor categories	Frequency	Proportion	95% CI	OMIM	ICD-10
***Neurological disorders***	**375**	**0.352**	**0.323-0.381**		
Intellectual disability (undefined)	61	0.057	0.043-0.071	249500	F79
Cerebral palsy	62	0.058	0.044-0.072	612900	G80.9
Intellectual disability, mild	57	0.054	0.040-0.067	300419	F70
Intellectual disability, moderate	47	0.044	0.032-0.056	300419	F71
Intellectual disability, severe/profound	34	0.032	0.021-0.042	300419	F72
Down syndrome	30	0.028	0.018-0.038	190685	Q90
Microcephaly	28	0.026	0.017-0.036	608716	Q02
Epilepsy	15	0.014	0.007-0.021	604364	G40
Global developmental delay	9	0.008	0.003-0.014	618330	Z13.42
Hypotonia, limbs	9	0.008	0.003-0.014	300868	P94.2
Spina bifida	8	0.008	0.002-0.013	182940	Q76.0
Macrocephaly	7	0.007	0.002-0.011	153470	Q75.3
Astasia	4	0.004	0.000-0.007		F44.6
Multiple sclerosis	2	0.002	-0.001-0.004	126200	G35
Canavan disease	1	0.001	-0.001-0.003	271900	E75.2
Tremor, congenital	1	0.001	-0.001-0.003	190300	R25.1
***Limb defects***	**281**	**0.264**	**0.237-0.290**		
Polydactyly, postaxial	53	0.050	0.037-0.063	174200	Q69
Talipes (types)	48	0.045	0.033-0.058	119800	Q66.6
Syndactyly (types)	45	0.042	0.030-0.054	610713	Q70.9
Limb deficiency disorders	39	0.037	0.025-0.048	217100	Q79.8
Polydactyly, preaxial	38	0.036	0.025-0.047	174400	Q69.0
Brachydactyly	21	0.020	0.011-0.028	112500	Q69.1
Leg length discrepancy	9	0.008	0.003-0.014		M21.7
Clinodactyly	8	0.008	0.002-0.013	148520	Q74.0
Camptodactyly	7	0.007	0.002-0.011	114200	Q68.1
Overriding toes	6	0.006	0.001-0.010		Q66.89
Split-hand/split-foot	2	0.002	-0.001-0.004	183600	Q72.7
Extra phalangeal joint, 5th finger	1	0.001	-0.001-0.003		
Megalodactyly/macrodactyly	1	0.001	-0.001-0.003	155500	Q74.2
Metaphyseal acroscyphodysplasia	1	0.001	-0.001-0.003	250215	
Toe contracture	1	0.001	-0.001-0.003		M24.5
Trigger thumb	1	0.001	-0.001-0.003	190410	M65.319
***Sensorineural defects***	**128**	**0.120**	**0.101-0.140**		
Deaf-mute	78	0.073	0.058-0.089	304500	H90
Mute only	27	0.025	0.016-0.035		F94.0
Microtia	9	0.008	0.003-0.014	600674	Q17.2
Stuttering	9	0.008	0.003-0.014	614668	F98.5
Deaf (mild)	5	0.005	0.001-0.009		H91
***Musculoskeletal defects***	**84**	**0.079**	**0.063-0.095**		
Dwarfisms/achondroplasia	18	0.017	0.009-0.025	100800	E34.3
Arthrogryposis	17	0.016	0.008-0.023	108120	Q68.8
Muscular dystrophy	17	0.016	0.008-0.023	310200	G71.0
Kyphosis	7	0.007	0.002-0.011	192900	Q76.4
Scoliosis	5	0.005	0.001-0.009	181800	M41.9
Kyphoscoliosis	4	0.004	0.000-0.007	610170	M41
Joint hypermobility	2	0.002	-0.001-0.004	147900	
Limb girdle muscular dystrophy	2	0.002	-0.001-0.004	253600	G71.0
Torticollis	2	0.002	-0.001-0.004	189600	M43.6
Juvenile rheumatoid arthritis	2	0.002	-0.001-0.004	180300	M06
Chondrodysplasia Grebe type	1	0.001	-0.001-0.003	228900	
Ellis-van Creveld syndrome	1	0.001	-0.001-0.003	225500	Q77.6
Exostosis	1	0.001	-0.001-0.003	133700	Q78.6
Gorham-Stout syndrome	1	0.001	-0.001-0.003		M89.9
Hyperflexibility of pectoral girdle	1	0.001	-0.001-0.003	169550	
Morquio syndrome	1	0.001	-0.001-0.003	309900	E76
Pectus excavatum	1	0.001	-0.001-0.003	169300	Q67.6
Spondylo-arthropathies	1	0.001	-0.001-0.003	106300	
***Visual impairments***	**67**	**0.063**	**0.048-0.077**		
Squint eye/strabismus	33	0.031	0.021-0.041	185100	
Blindness	22	0.021	0.012-0.029	216900	H53.5
High myopia	4	0.004	0.000-0.007	160700	H52.10
Ptosis	4	0.004	0.000-0.007	300245	Q10.0
Night blindness	2	0.002	-0.001-0.004	310500	H53.60
Anophthalmia	1	0.001	-0.001-0.003	251600	Q11.2
Ophthalmoplegia	1	0.001	-0.001-0.003	605637	
***Hemoglobinopathies***	**40**	**0.038**	**0.026-0.049**		
Thalassemia	35	0.033	0.022-0.044	613985	D56
Haemophilia	5	0.005	0.001-0.009	306700	D66
***Ectodermal disorders***	**34**	**0.032**	**0.021-0.042**		
Albinism	12	0.011	0.005-0.018	203100	E70.3
Ichthyosis	3	0.003	0.000-0.006	242300	Q80.9
Alopecia areata	2	0.002	-0.001-0.004	104000	L63
Alopecia universalis	2	0.002	-0.001-0.004	203655	L63.1
Atopic eczema	2	0.002	-0.001-0.004	603165	L20
Epidermolysis bullosa dystrophica	2	0.002	-0.001-0.004	226600	Q81.2
Palmoplantar keratoderma	2	0.002	-0.001-0.004	144200	L40.3
Anonychia	1	0.001	-0.001-0.003	161050	L60.3
Fibromatosis	1	0.001	-0.001-0.003		M72.30
Hyperhidrosis	1	0.001	-0.001-0.003		R61
Hypertrichosis	1	0.001	-0.001-0.003	135400	
Knuckle pads	1	0.001	-0.001-0.003	149100	M72.1
Oligodontia	1	0.001	-0.001-0.003	106600	K00.0
Peeling skin syndrome	1	0.001	-0.001-0.003	270300	
Polythelia	1	0.001	-0.001-0.003	163700	Q83.1
Psoriasis	1	0.001	-0.001-0.003	605606	L40
***Cardiovascular anomalies***	**19**	**0.018**	**0.010-0.026**		
Ventricular septal defect	13	0.012	0.006-0.019	614429	Q21.0
Arterial septal defect	4	0.004	0.000-0.007	108800	Q21.1
Fallot’s Tetralogy	2	0.002	-0.001-0.004	187500	Q21.3
***Orofacial anomalies***	**19**	**0.018**	**0.010-0.026**		
Cleft lip and palate	12	0.011	0.005-0.018	119530	Q37.0
Cleft lip	4	0.004	0.000-0.007	119540	Q35
Ankyloglossia	1	0.001	-0.001-0.003	106280	Q38.1
Deviated nasal septum	1	0.001	-0.001-0.003		J34.2
Gingival fibromatosis	1	0.001	-0.001-0.003	135300	K06.1
***Others***	**18**	**0.017**	**0.009-0.025**		
Gut atresia congenital	2	0.002	-0.001-0.004		Q41.1
Hypospadias	2	0.002	-0.001-0.004	300633	Q54.9
Bardet-Biedl syndrome	1	0.001	-0.001-0.003	209900	Q87.8
Congenital aphallia	1	0.001	-0.001-0.003		Q55.5
Congenital urethrorectal fistula	1	0.001	-0.001-0.003	314390	N36.0
Criger-Najjar syndrome	1	0.001	-0.001-0.003	218800	
Cryptorchidism	1	0.001	-0.001-0.003	219050	Q53.1
Cystic fibrosis	1	0.001	-0.001-0.003	603855	
Diabetes type-I	1	0.001	-0.001-0.003	222100	P70.2
Ectopic vesicae	1	0.001	-0.001-0.003		Q64.10
Graves disease	1	0.001	-0.001-0.003	608173	
Hernia, navel protruding	1	0.001	-0.001-0.003		K40-K46
Lymphedema	1	0.001	-0.001-0.003	153100	Q82.0
Minimal change disease	1	0.001	-0.001-0.003		N05.0
Neurogenic bladder	1	0.001	-0.001-0.003	600057	Q64.1
Wilson’s disease	1	0.001	-0.001-0.003	277900	E83.01

Among the neurological disorders, there were at least 16 distinct entities, and among those intellectual disabilities were most conspicuous and collectively involved 24% of the subjects (including Down syndrome and microcephaly, but not including cerebral palsy (CP)) (Table-II). The CP was evident in 5.8% of subjects. In the limb defects, there were 16 distinct subtypes most prominent of which were polydactyly (postaxial), followed by talipes and syndactyly. Among the sensorineural defects, the subjects with deaf-mute were prominent (7.3%). In the musculoskeletal defects, there were 18 distinct entities and among those dwarfisms were most common (1.7%), followed by arthrogryposis and muscular dystrophy. In visual impairments, the most common anomaly was squint eye/strabismus (3.1%), followed by blindness.

In the category of hemoglobinopathies, thalassemia had a common presentation (3.3%) while there were minor cases of haemophilia (0.5%) (Table-II). In ectodermal disorders, there were 16 distinct entities and among those, albinism was most conspicuous (1.1%). Among the cardiovascular anomalies, the common presentation was ventricular septal defects (1.2%), followed by arterial septal defects and Fallot’s tetralogy. Among the orofacial anomalies, there were five distinct entities and cleft lip and palate were most prominent (1.1%). The last category ‘others’ comprised 16 distinct entities. It comprised rare disorders which could not be classified into the above main categories.

### Familial vs. sporadic presentation:

The majority of the malformations had a sporadic presentation, n=721 (68%), whereas 32% of cases had familial occurrences (Table-III). The highest representation of familial cases was observed in hemoglobinopathies and ectodermal disorders (63% and 62%, respectively). On the other hand, the cardiovascular anomalies, orofacial anomalies and musculoskeletal defects had sporadic appearances most often (95%, 84%, and 74%, respectively).

**Table-III:** Familial vs. sporadic occurrence and isolated vs. syndromic presentation in major categories of CA.

Major category	No.	Familial sporadic occurrence (No. %)		Isolated/ syndromic presentation (No. %)		Total affected family members		

		Sporadic	Familial	Isolated	Syndromic	All families (n=1065)	Familial cases (n=344)	Sporadic cases (n=721)
Neurological disorders	375	258 (69)	117 (31)	115 (31)	260 (69)	583	325	258
Limb defects	281	203 (72)	78 (28)	275 (98)	6 (2)	459	256	203
Sensorineural defects	128	85 (66)	43 (34)	115 (90)	13 (10)	210	125	85
Musculoskeletal defects	84	62 (74)	22 (26)	75 (89)	9 (11)	132	70	62
Visual impairments	67	37 (55)	30 (45)	62 (93)	5 (7)	158	121	37
Hemoglobinopathies	40	15 (38)	25 (63)	39 (98)	1 (3)	88	73	15
Ectodermal disorders	34	13 (38)	21 (62)	32 (94)	2 (6)	107	94	13
Cardiovascular anomalies	19	18 (95)	1 (5)	18 (95)	1 (5)	30	12	18
Orofacial anomalies	19	16 (84)	3 (16)	18 (95)	1 (5)	28	12	16
Others	18	14 (78)	4 (22)	17 (94)	1 (6)	25	11	14
Total	1,065	721 (68)	344 (32)	766 (72)	299 (28)	1820	1099	721
		Chi2=48.89; p<0.0001		Chi2=492.3; p<0.0001				

### Isolated vs. syndromic occurrence:

In this cohort, most of the anomalies had isolated appearance, n=766 (72%) and syndromic occurrence were observed in 28% of the cases (Table-III). The isolated occurrence was common in all major categories except neurological disorders which had a preponderance of syndromic cases (69%). The differences in the distribution of major categories with respect to isolated/syndromic occurrence were statistically highly significant (p<0.0001).

In all 1065 families, there were a total of 1820 subjects affected (males 1191; females 629). Among the familial cases alone, the number of affected subjects was 1099 compared to 721 sporadic cases (Table-III).

## DISCUSSION

A national disease registry for CA does not exist in Pakistan. This is the first record of CA prevalent in the pediatric and adult population of Peshawar Valley of Pakistan. Population-based studies on CA in the adult strata are difficult to conduct due to lack of awareness, poor consent approval and language and logistic issues in the rural and socio-economically deprived populations.^15^,^18^-^19^ Like other resource deficient countries, in Pakistan the rural areas have an inadequate infrastructure of health facilities and prenatal screening, antenatal care and medical genetic services are deficient.^15^ Various genetically determined anomalies like haemoglobinopathies, metabolic disorders and enzymopathies were previously very common in certain countries of the Mediterranean region; however, due to the advancement of genetic services the prevalence of these disorders has drastically reduced.

There was a preponderance of index males compared to the index females (71% vs. 29%, respectively). Gender disparities in the incidence of CA have been witnessed in many studies. Researchers have witnessed that certain anomalies like polydactyly and cleft lip tend to be more common in males, whereas neural tube defects and cleft palate are more prevalent in females.^3^,^20^

In the present cohort, there was the highest representation of neurological disorders, followed by limb defects, sensorineural defects, and musculoskeletal defects. Concordantly, in a sample of CA ascertained by Zahra et al. from Kurram Agency of Pakistan, the most frequent anomalies were neurological disorders, followed by musculoskeletal defects, limb anomalies and sensorineural defects.^11^ However, in the experience of Bhatti et al., in a cohort assembled from the Sialkot district of Pakistan, the largest representation was limb defects followed by neurological disorders.^10^ Ullah et al. demonstrated that among the limb defects in the population of Chitral, Pakistan, polydactyly was very common followed by syndactyly and absence deformities.^12^ Hence, the prevalence-pattern of CA varies across populations.

In a cohort of neonates studied at the Khyber teaching hospital in Peshawar, Khan et al. observed that neurological disorders were most common and among those Hydrocephaly (23%), anencephaly (13%), and spina bifida (10%) were major anomalies.^21^ Here, hemoglobinopathies and ectodermal disorders had the highest ratio of familial cases (63% and 62%, respectively). In the present data, pedigree analyses of those cases revealed segregation of malformations in multiple generations and multiple sibships (data not shown). In most of the families, the autosomal recessive inheritance pattern was obvious. It is pertinent to mention that sporadic cases were defined as single affected subjects in the family with no family history of that anomaly, whereas familial cases had at least two affected subjects with similar phenotypic presentation. However, it is quite likely that few of the sporadic cases may have familial nature.

### Strengths of study:

This study has several strengths. Firstly, it focuses on the pediatric and adult populations, contrasting to the previous studies which were hospital-based and reported CA among the neonatal population.^17^,^18^ Secondly, this study employs large first-hand data of 1065 independent subjects. To the best of our knowledge, no such large data on CA have been reported from the general populations of Pakistan.^14^-^16^,^21^ Thirdly, this study reports a large spectrum of disorders, and at least 104 distinct entities were presented. The data presented here could be a way forward to a national disease registry database.

### Limitations:

This study has several limitations. For instance, the true prevalence and incidence of CA are not reported. Further, the ethnic and linguistic differentials in the prevalence of CA is not presented. The underlying etiological factors for CA were not explored. The sample may be biased toward the CA which have clearcut phenotypes and have non-lethal nature. Nonetheless, the role of parental consanguinity among the major and minor CA needs to be elucidated. In a prospective study, it would be interesting to focus on maternal factors like the effect of diet and drugs, environmental exposures, pregnancy events and delivery/neonatal factors (reviewed in Lee et al.^7^).

## CONCLUSION

In conclusion, this is a pilot record of CA in the pediatric and adult strata of the Peshawar population of Pakistan. A wide range of CA were witnessed in this cohort with a preponderance of neurological disorders. The majority of the anomalies are of severe nature rendering high morbidity burden and requiring early detection and intervention. The key findings of this study could be useful in the management, resource allocation and policy making for CA.

### Authors Contribution:

**SM:** Conceived, designed and supervised the study.

**SFN, UM, WUQ, SA** and **AI:** Data collection and Statistical analysis.

**SM:** Responsible and accountable for the accuracy and integrity of data.
